# Mouse Ribosomal RNA Genes Contain Multiple Differentially Regulated Variants

**DOI:** 10.1371/journal.pone.0001843

**Published:** 2008-03-26

**Authors:** Hung Tseng, Weichin Chou, Junwen Wang, Xiaohong Zhang, Shengliang Zhang, Richard M. Schultz

**Affiliations:** 1 Department of Dermatology, University of Pennsylvania, Philadelphia, Pennsylvania, United States of America; 2 Cell and Developmental Biology, University of Pennsylvania, Philadelphia, Pennsylvania, United States of America; 3 Center for Research on Reproduction and Women's Health, University of Pennsylvania, Philadelphia, Pennsylvania, United States of America; 4 Center for Bioinformatics, Department of Genetics, University of Pennsylvania, Philadelphia, Pennsylvania, United States of America; 5 Department of Computer and Information Science, University of Pennsylvania, Philadelphia, Pennsylvania, United States of America; 6 Department of Biology, University of Pennsylvania, Philadelphia, Pennsylvania, United States of America; The Babraham Institute, United Kingdom

## Abstract

Previous cytogenetic studies suggest that various rDNA chromosomal loci are not equally active in different cell types. Consistent with this variability, rDNA polymorphism is well documented in human and mouse. However, attempts to identify molecularly rDNA variant types, which are regulated individually (i.e., independent of other rDNA variants) and tissue-specifically, have not been successful. We report here the molecular cloning and characterization of seven mouse rDNA variants (v-rDNA). The identification of these v-rDNAs was based on restriction fragment length polymorphisms (RFLPs), which are conserved among individuals and mouse strains. The total copy number of the identified variants is less than 100 and the copy number of each individual variant ranges from 4 to 15. Sequence analysis of the cloned v-rDNA identified variant-specific single nucleotide polymorphisms (SNPs) in the transcribed region. These SNPs were used to develop a set of variant-specific PCR assays, which permitted analysis of the v-rDNAs' expression profiles in various tissues. These profiles show that three v-rDNAs are expressed in all tissues (constitutively active), two are expressed in some tissues (selectively active), and two are not expressed (silent). These expression profiles were observed in six individuals from three mouse strains, suggesting the pattern is not randomly determined. Thus, the mouse rDNA array likely consists of genetically distinct variants, and some are regulated tissue-specifically. Our results provide the first molecular evidence for cell-type-specific regulation of a subset of rDNA.

## Introduction

Mammalian ribosomal RNA genes are comprised of several hundreds of transcription units clustered on a number of chromosomal loci [1], [2]. Cytogenetic studies showed that in human, individual chromosomal rDNA loci were not equally active in different cell types [3], [4]. A similar observation was also made with plant cells [5]. These studies raised the possibility of the existence of regulatory sub-domains in the rDNA array and their cell-type-specific regulation (for a review [6]).

Polymorphic variations in rDNA are well documented [7]–[15]. In mouse, restriction fragment length polymorphism (RFLP) was noted in the 5′-end of the rDNA unit and attributed to a variable number of repeats in the non-transcribed spacer [16]. These RFLPs belong to 2–3 independent linkage groups, which are distributed on several chromosomes and stable among mouse strains (i.e., inter-group sequence exchange is rare) [8], [16], [17]. However, attempts to identify subsets of rDNA, which are regulated differentially among tissues, have not been successful. Six polymorphisms in human 28S rRNA V5 region were identified and used as markers for individual rDNA genes to investigate their expression in different tissues, but no consistent tissue-specific expression pattern was observed (e.g., [18]). The ability of identifying rDNA variants is also hampered by the lack of genomic sequence information of both human and mouse rDNA loci (e.g., in GenBank Release 163, December, 2007, only one mouse rDNA transcription unit has been sequenced in its entirety, i.e., [19]). This lack of sequence information precludes employing computational and bioinformatic methods to identify rDNA variants.

Another not-well-explored area in the regulation of rRNA synthesis is its cell-type-specificity [6]. In multicellular organisms, because of differentiation of cellular functions, some cells may have different requirements for rRNA synthesis than others. Cell-type-specific regulation of rRNA synthesis was first witnessed during *Xenopus* oogenesis, in which the rDNA array was amplified several thousand-fold to boost rRNA synthesis [20], [21]. This amplification is achieved by a rolling-circle mechanism, which is both cell-type- and developmental-stage-specific [22].

Our recent study of basonuclin suggests that rRNA transcription is modulated by cell-type-specific factors [23]–[25]. Basonuclin (BNC1, *Bnc1*, MGI) is a zinc finger protein with highly restricted cell-type distribution–it is mainly expressed in keratinocytes and the reproductive germ cells. BNC1 interacts with rDNA promoter and influences Pol I transcription (for reviews, [26], [27]). We reported that in BNC1-deficient oocytes only a subset of Pol I transcription foci was affected [23]. The selective effect of BNC1-deficiency on Pol I foci suggests that cell-type-specific rRNA regulation relates to usage of subsets of rDNA. This notion led us to search for rDNA variants (v-rDNA). Here we report the molecular cloning of seven v-rDNAs, and the characterization of their copy numbers, sequence, epigenetic modification, and expression profiles in multiple tissues.

## Results

### Cloning RFLP of the mouse rDNA

**Figure 1:**
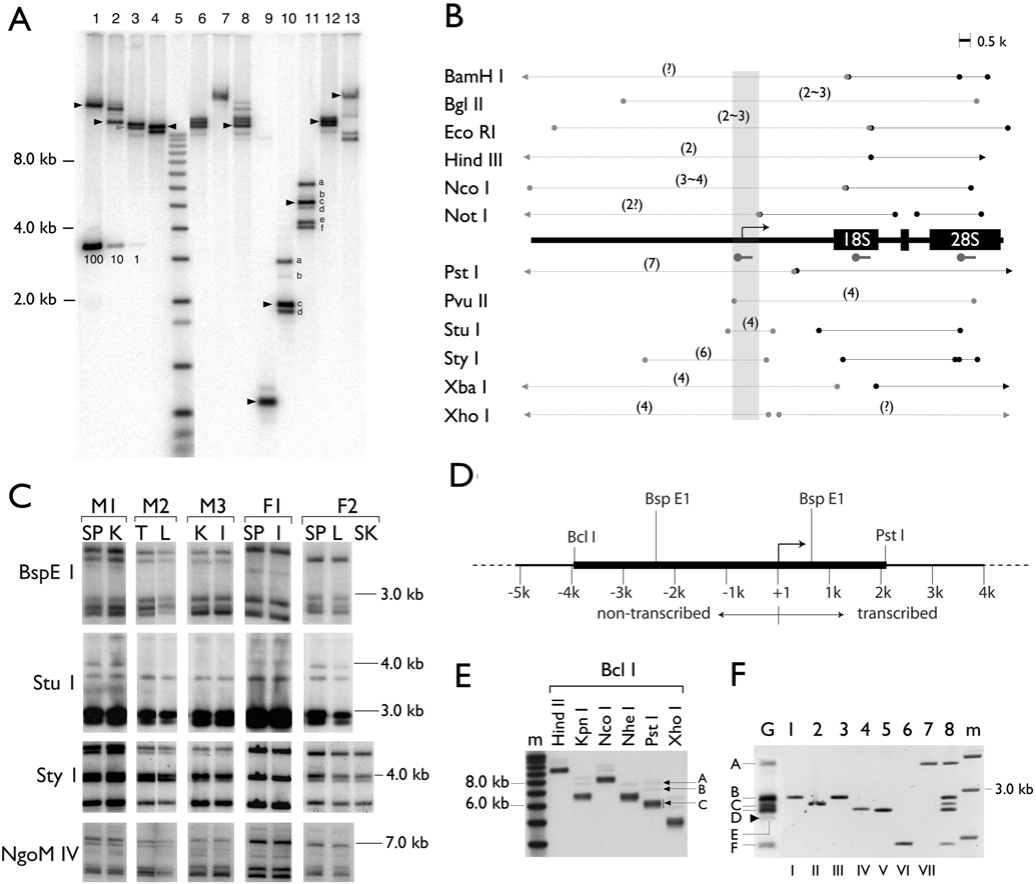
Molecular cloning of rDNA variants from CF1 mouse. A, An example of rDNA RFLPs revealed by Southern analyses of CF1 liver genomic DNA. The Southern blot was probed with a DNA fragment from the promoter-leader region of rDNA. A molecular weight marker (mw) is included (lane 5) and the molecular weights indicated to the left. Bands of size predicted by the published rDNA sequence (BX000964) are indicated by arrowheads. A serial copy number marker is indicated by the copy numbers each band contains (i.e., 100, 10, 1). The copy number was calculated as described in Tian et al., 2001, using 2.7×10^9^ bp for the haploid mouse genome. The fragments, whose copy numbers were assessed and described in the text, are alphabetically labeled (lanes 10 and 11). The restriction enzymes used to generate A are the most informative among the thirty enzymes tested. They are listed as they appear in B (i.e., lane 1, BamHI, lane 2, Bgl II, with the exception of lane 5). B, A summary of the RFLPs detected by the Southern analyses. A generic rDNA (partial) is depicted in the middle, with transcription start site (bent arrow), the coding sequence (18S and 28S) and the hybridization probe (hammers) indicated. The polymorphism or lack thereof is depicted by circle-ended grey lines and black lines, respectively. The number of polymorphisms for a given restriction fragment is shown in parentheses. The gray area indicates the variable region around the transcription start site. C, The promoter region RFLP is stable during organ development. Genomic DNA was isolated from tissues of five unrelated mice, three males (M1, 2 and 3) and two females (F1 and 2) and analyzed by Southern blots. Tissue genomic DNAs were digested with one of the four enzymes (Bsp E1, Stu I, Sty I and NgoM IV) as indicated to the left of the gel image. The blots were probed with the same rDNA promoter fragment as shown in A. SP, spleen, K, kidney, T, testis, L, liver, I, intestine, SK, skin. Note that no variation is detected among the tissues of an individual. D, A restriction map indicates the cutting sites of relevant enzymes. The thicker line depicts the target fragment for cloning. The indicated size (∼6 kb) is that predicted by the published sequence. E, Liver genomic DNA was double-digested with Bcl I and another restriction enzyme and probed with the promoter-leader probe. Bcl I-Pst I digestion yielded three groups of bands, which were purified individually by size-fractionation and used to generate libraries designated A, B and C, respectively. F, Seven variant rDNAs (v-rDNA) were cloned and each contained an internal BspE1 fragment (lanes 1 to 7) that matched a BspE1 RFLP of the genomic DNA (lane G). When mixed as a group, the BspE1 digestion pattern of these v-rDNA clones (lane 8) resembles that of the genomic digestion (lane G). Note one v-rDNA RFLP was not isolated (band E, arrow head in lane G).

The RFLPs in the non-transcribed spacer region of rDNA were noted three decades ago [8], [9], [28]. To identify rDNA RFLPs that could be used in isolating potential rDNA variants (v-rDNA), we examined the RFLPs of thirty restriction enzymes for their size distribution and stability during organogenesis. For size distribution analysis, mouse liver genomic DNA (Strain CF1) was cut in single or double restriction digestions and subjected to Southern analyses (**Fig. 1A**). Three hybridization probes were used to examine 1) the promoter and the transcript-leader region (referred to as promoter-leader), 2) the 18S, and 3) 28S rRNA coding regions (**Fig. 1B**). Some restriction digestions were not informative (i.e., they cut at either too many or too few sites). Only the informative digestions are shown in Fig. 1. In general, the promoter-leader region contained many RFLPs (**Fig. 1A**) and the number of RFLPs varied from two to seven (e.g., **Fig. 1A** lanes 4 and 8, respectively). In contrast, the rRNA coding regions (18S and 28S) contained no RFLP, i.e., the fragment size predicted by the known sequence (BK000964) agreed well with the size of the bands (Southern data not shown but summarized in **Fig. 1B**, black lines). These Southern analyses confirmed previous conclusions that most of the RFLPs were near the transcription start site or up-stream from it (**Fig. 1B**, gray area).

The rDNA RFLPs were stable among individuals of inbred strains [17]. However, the stability of the RFLPs within an individual (i.e., during development and organogenesis) had not been examined before. Accordingly, genomic DNA was isolated from various tissues (spleen, kidney, testis, liver, intestine and skin) of five unrelated CF1 mice (two females and three males) and analyzed by Southern blots (**Fig. 1C**). To ensure sensitivity in detecting variations, each of the four restriction enzymes used for the Southern analysis produced at least four RFLPs. This analysis showed no RFLP variability at the rDNA promoter among tissues of the same individual, suggesting no recombination among rDNA RFLPs during organogenesis. Taken together, our results agreed well with a previous study [17] that rDNA recombination among non-homologous chromosomes is rare and that rDNA contains genetically stable variants (v-rDNA).

To identify and characterize sequence variations that were uniquely associated with each type of v-rDNA, we identified a Bcl I-Pst I fragment that contained the transcription start site (TSS) and the surrounding 6 kb sequence (**Fig. 1D**). Southern analysis showed that in the CF1 mouse strain there were at least three groups (A, B and C) of the Bcl I-Pst I RFLPs ranging from 6.2 to 7.8 kb (**Fig. 1E**). To clone these RFLPs, the Bcl I/Pst I-digested CF1 genomic DNA was size-fractioned on an agarose gel. From the bands indicated in **Fig. 1E**, three libraries (A, B, C) were constructed, which were then screened with a promoter-leader probe by colony hybridization. Five, 8 and 20 positive clones were identified from libraries A (8.0 kb), B (7.3 kb) and C (6.0 kb), respectively. Restriction analysis by four enzymes (Bsp E1, Bsr GI, Pvu II and Stu I) revealed that these 33 cloned RFLPs belonged to seven distinct types of v-rDNA, named Type I through VII (**Table 1**). Types I through V were isolated from library C, VI from A, VII from B. To assess how well the genomic RFLP was represented by the cloned v-rDNA, each clone was digested with Bsp EI, which generated an internal fragment (**Fig. 1D**), and the resulting pattern was compared with that of the genomic Bsp EI RFLP in Southern analyses (**Fig. 1F**, Lane G). The results showed that the cloned rDNA corresponded well to the genomic RFLP, with the exception of one band with low copy number (**Fig. 1F**, Lane G, arrowhead).

**Table 1:** Classification the rDNA variants.

	Bsp E1^1^ (kb)	BsrG1^2^	Pvu II^3^ (kb)	Stu I^4^
I	2.9	+	0.60	-
II	2.8	+	0.60	-
III	2.9	-	0.75	+
IV	2.6	-	0.60	-
V	2.6	+	0.60	-
VI	1.9	+	0.60	+
VII	3.9	-	0.75	+

1. Length of the Bsp E1 fragment containing the transcription initiation site (+1). See also Fig. 1D.

2. The presence (+) or absence (−) of the BsrG1 site at −519.

3. The length of the Pvu II fragment containing the transcription initiation site.

4. A Stu I site at −1200, which is not present in the previous published sequence (BK000964).

### Copy number of mouse v-rDNA

To assess the size of the rDNA pool detected by our Southern analysis, we measured the copy numbers of the Stu I and Sty I RFLPs using a set of markers (**Fig. 1A** lanes 1, 2 and 3, representing 100, 10 and 1 copies, respectively). The copy number of Stu I RFLPs (**Fig. 1A**, Lane 10) was: a, 19; b, 2; c, 22; d, 12 (a total of 55 copies). Similarly, the copy-number of Sty I RFLPs (**Fig. 1A**, Lane 11) was: a, 14; b, 7; c, 21; d, 11; e, 16; and f, 14 or a total of 83 copies. This analysis suggested we were examining an rDNA pool of less than 100 copies, which fell short of the estimated several hundred copies of rDNA in the mammals. Thus we were likely dealing with a subset of the total rDNA complement.

**Figure 2:**
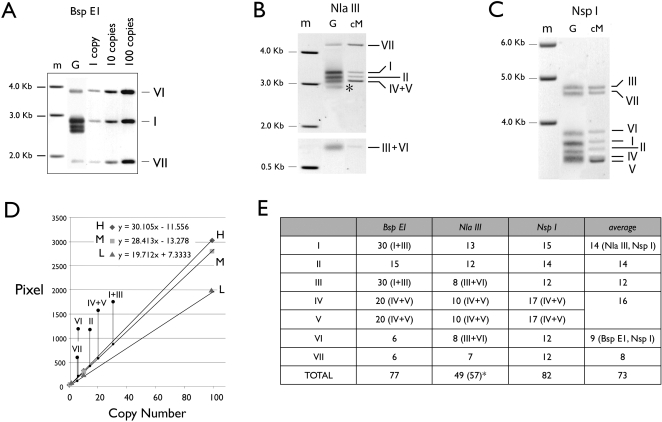
Copy numbers of v-rDNA. Measurements are based on RFLP of three restriction enzymes, Bsp E1 (A), Nla III (B) and Nsp I (C). The genomic fragment of each v-rDNA was identified by its clone digested with the same restriction enzyme (e.g., Fig. 1F) and the identity is indicated on the right of each Southern image. In A, copy number markers (1, 10 and 100 copies) of high (v-rDNA VI), medium (v-rDNA I) and low (v-rDNA VII) molecular weights were included on the right of the genomic DNA (G) In B and C, the identification markers, which are generated by digesting a mixture of cloned v-rDNA (cM), are shown to the right of the genomic DNA. m, molecular weight markers. D shows, as an example, the quantification of Bsp E1 RFLP. Three standard curves and their mathematical descriptions are shown; H, high-, M, medium- and L, low-molecular weight range. The density measurement of each RFLP band is mapped onto the standard curve of the appropriate molecular weight range. E, A summary of the copy number measurements. The copy numbers are rounded to the next integer. The Roman numerals in the parenthesis indicate the RFLPs that cannot be separated by gel electrophoresis. The average copy number was calculated from unambiguously identified RFLPs only. For the Nla III digestion, one RFLP had no corresponding v-rDNA clone (*). The total copy number of Nla III RFLP is estimated without this RFLP (i.e., 49) or with (i.e., 57).

With the aid of the cloned v-rDNA fragments the genomic copy number of each v-rDNA type was assessed. The assessment was based on our ability to match the genomic RFLP with the restriction fragment produced by each clone (**Fig. 1F**, **Fig. 2A, B, C**). We screened twelve restriction enzymes that cut internally within the cloned v-rDNA fragments and selected three (Bsp E1; Nla III and Nsp I) for this analysis (**Fig. 2A, B, C**). No restriction enzyme digestion could separate v-rDNA IV and V and they were measured together. To increase the accuracy of the measurement, copy number markers of different molecular weight (**Fig. 2A**) were included in the Southern analysis to control for the variable diffusion rate of different fragment size in the gel, which resulted in a faster loss of smaller DNA from the gel during electrophoresis. Quantification of the genomic RFLPs was based on three standard curves covering the high, medium, and low molecular weight ranges of the RFLPs (**Fig. 1D**). The copy number analyses (**Fig. 1E**) demonstrated that all variant types were present in similar copy numbers and that the total copy number of the rDNA pool analyzed by this method was between 49 to 82, in agreement with the Stu I- and Sty I-RFLP copy number analyses (**Fig. 1A**).

### Sequence of v-rDNA

**Figure 3:**
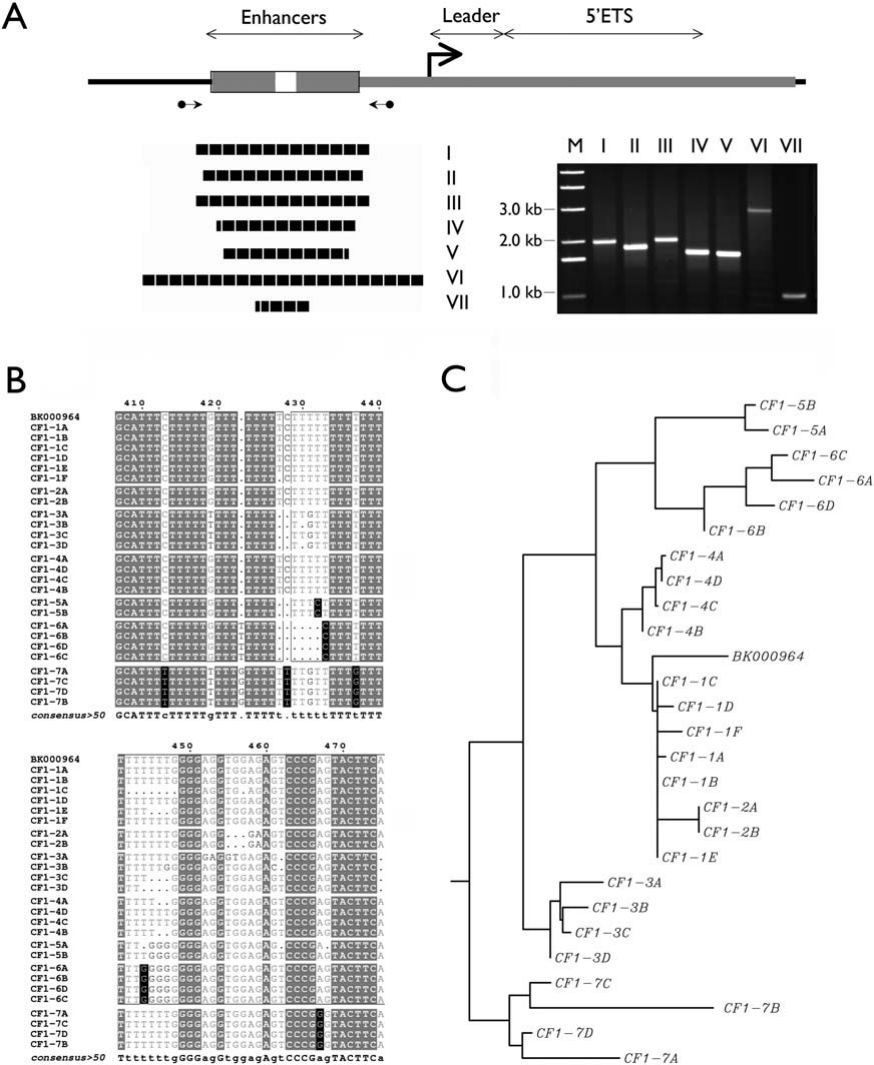
Structure and sequence variation in v-rDNA. A, A generic rDNA promoter is depicted at the top, showing the transcription start site (bent arrow), the enhancer (boxes), the 5′ external transcribed spacer (ETS) and its leader. Gray area represents the region sequenced. DNA sequencing and PCR analysis showed that the RFLPs around the promoter are due, in part, to the variable size of the enhancer. Because of uncertainties associated with aligning repetitive sequence, the size of the enhancer region was verified by PCR with primers flanking the region (circle-ended arrows). The resulting PCR fragments are shown in the gel panel, in which the templates (cloned v-rDNA) are indicated above each lane. The number of repeats in each enhancer region was estimated (black blocks, each represents one unit). Some units are partial as evidence in their DNA sequence. B, Multi-sequence alignment reveals variant-specific SNPs in the transcribed region (5′-ETS). The sequences (+1 to +2000) of the 26 v-rDNA clones are aligned with the known rDNA sequence BK000964. Only a segment of the alignment (+407 to +475) is shown as an example. The nucleotides identical to all v-rDNAs are displayed as white letters in a gray background, the variable nucleotides as gray letters in a white background and the variant-specific nucleotide as white letters in a black background. Listed at the bottom of the alignment is the consensus sequence. C, The same alignment is displayed as a dendrogram to show the genetic distance among the v-rDNA types.

A total of 26 v-rDNA clones were sequenced, representing approximately one third (26/73) of the estimated copies of the rDNA pool studied here, i.e., individually, for I+II, 29% (8/28); III, 33% (4/12); IV, 33% (4/12); V, 50% (2/4); VI, 44% (4/9) and VII, 50% (4/8) )(Genbank accession numbers are given in Table S1. Sequencing more clones would likely risk repeat sampling. The promoter and transcribed regions were sequenced in their entirety. The enhancer region was sequenced from both ends and due to priming difficulty in repetitive sequence, a gap of 100–200 bp was left at the center of the enhancer region (**Fig. 3A**). These sequences confirmed that v-rDNAs had an enhancer region of variable size (**Fig. 3A**), which was the main cause of RFLPs in the promoter region [17], [29]–[32]. Within each enhancer region, the number of repeat units varied from 4 to 20, which was estimated from the sequence and the size of the PCR amplicon of the enhancer region (**Fig. 3A**). Multi-sequence alignment analysis of the transcribed region (+1 to +2000) revealed single nucleotide polymorphisms (SNPs), some of which were variant-specific and could be used to distinguish the transcript from each v-rDNA type (**Fig. 3B**). The sequence alignment also confirmed most of the classification of the v-rDNA and delineated the relationships among the types (**Fig. 3C**). Type I and II v-rDNAs were apparently very closely related and indistinguishable from each other (**Fig. 3C**). These two types were also better related to the known rDNA sequence (BK000964) than the other v-rDNAs (**Fig. 3C**).

### Genetic and Epigenetic variations in the v-rDNA promoter

**Figure 4:**
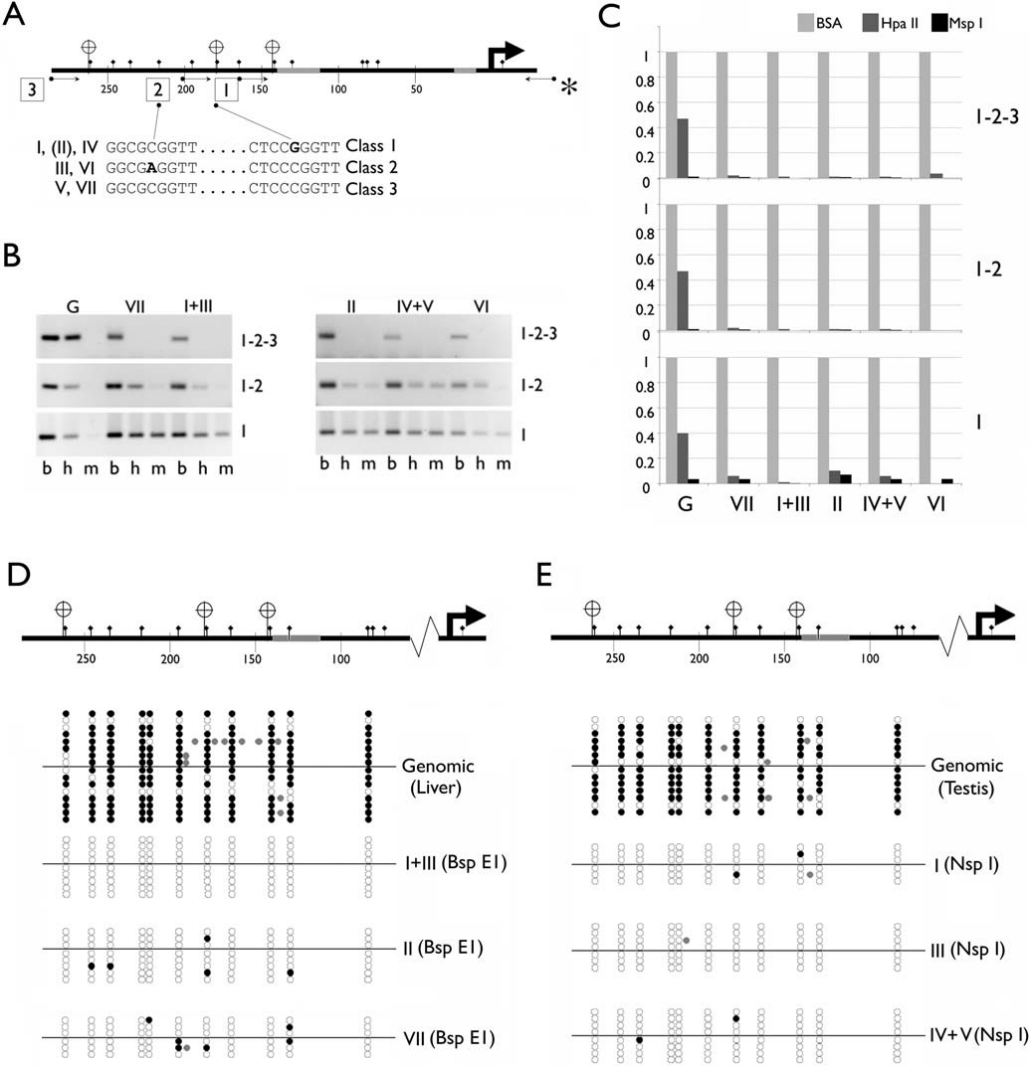
SNPs and DNA methylation of the v-rDNA promoters. A, Two SNPs define three classes of rDNA promoter. A generic rDNA promoter is depicted at the top and the location of the SNPs (bold letters in the sequence) is shown below. The v-rDNAs associated with the SNP are indicated on the left of the sequence and the promoter classification on the right. Transcription start site (bent arrow), cis-elements (grey sections), HpaII sites (banners) and CpG islands (diamond-headed pins). B, The methylation status of the CpCpGpG was examined by Hpa II and Msp I digestions followed by PCR amplification of the fragment containing the cut site. Hpa II-resistance indicates methylation at the CpG within the site, where as MspI-resistance indicates CpC methylation. Three PCRs were designed to examine the Hpa II/Msp I sites at −144, −179 and −261 using primers indicated in (A). The PCR primers (circle-ended arrows) share an anchor primer (*) and the other primers determine whether the PCR examines one, two or three HpaII sites as indicated by the boxed number in (A). The number of Hpa II sites examined is indicated on the right of the gel panel in (B). The variant types examined are listed on top and restriction enzymes used below each lane. For each DNA sample, three digestions were performed. b, bovine serum albumin (no digestion control), h, Hpa II and m, Msp I. C, The results in (B) were verified by quantitative PCR (qPCR, n = 2), in which the quantity of amplified genomic sequence is assigned as 1.0. D and E, Cytosine-methylation at other sites was evaluated by bisulfite sequencing. Genomic DNA from liver (D) and testis (E) was digested by Bsp I (D) or Nsp I (E) and six v-rDNA types were isolated based on their co-migration with the cloned v-rDNA digested with the same restriction enzyme. The genomic DNA served as a reference. Randomly picked rDNA promoters were sequenced from either strands (above and below the line) and cytosine methylation data are aligned with a generic rDNA promoter depicted on the top of the panel. Black circle, methylated, open circle, unmethylated, gray circle, methylation of non-CpG (e.g., CpC sites).

Within the promoter region (+1 to −300) of the seven v-rDNAs, only two nucleotide substitutions were present (**Fig. 4A**), which classified the promoters into three classes. Compared with the consensus, Class 1 promoters (I, II and IV) contained at −179 a C to G transversion. Class 2 promoters (III and VI) contained a C to A transversion at −219. Class 3 was the consensus sequence. Interestingly, both transversions occurred within CpG sites. At −179, the C to G transversion shifted the CpG one nucleotide up-stream, whereas at −219, the C to A transversion abolished a CpG site. Such genetic variations prompted us to examine DNA methylation of the v-rDNA promoters.

Each of the seven v-rDNA promoters contained three HpaII/MspI sites (at −144, −179 and −261), which were used to assess the DNA methylation status of the promoters (**Fig. 4B, C, D, E**). The lack of sequence divergence within the v-rDNA promoters precluded the possibility of selectively PCR-amplifying each promoter via variant-specific primers. Instead, v-rDNA promoters were isolated by the size difference of their RFLP (**Fig. 1F** and **Fig. 2C**). Mouse liver and testis genomic DNAs were digested with Bsp E1 or Nsp I and electrophoresed alongside a v-rDNA clone digested by the same restriction enzyme. The cloned v-rDNA served as a marker for the mobility of their genomic counterpart. The promoter region of individual v-rDNA (or two when they could not be separated) was purified from the gel slice and digested with Hpa II or Msp I. The Hpa II/Msp I-sensitivity of each CpCpGpG was examined by PCR using primers amplifying one, two, or all three CpCpGpG sites (**Fig. 4A**). Consistent with previous reports, genome wide, about 50% of all rDNA promoters were Hpa II-resistant at all three sites, suggesting they were methylated at the CpG (**Fig. 4B**). Regular and quantitative PCR (qPCR) showed all seven v-rDNA were hypomethylated at all three Hpa II sites, and a slight increase in CpC methylation of Hpa II site at −144 (**Fig. 4C**). Bisulfite sequencing analysis confirmed that genomic DNA contained two types of rDNA promoters, one that was methylated at virtually all CpG sites and one that contained only a few methylated CpG sites (**Fig. 4D, E**, genomic). Notably, in the randomly selected genomic rDNA clones, on average only 17% (2/16 in liver and 3/14 in testis) were fully unmethylated, which was much lower than the value (∼50%) revealed by the HpaII/MspI sensitivity assay (**Fig. 4B**). Bisulfite sequencing analysis also showed hypomethylation at all CpG sites in the promoter of identified v-rDNAs in mouse liver (**Fig. 4D**) and testis (**Fig. 4E**). In these v-rDNA promoters only sporadic CpG methylation was seen. Also detected was methylation of several non-CpGs (**Fig. 4D and E**, gray circles), including CpC methylation, which was considered rare and only present in the embryonic stem cells [33]. These data suggested that the seven v-rDNAs belonged to the hypomethylated portion of the genomic rDNA complement and they were likely transcribed.

### Variant-specific PCR assays

**Figure 5:**
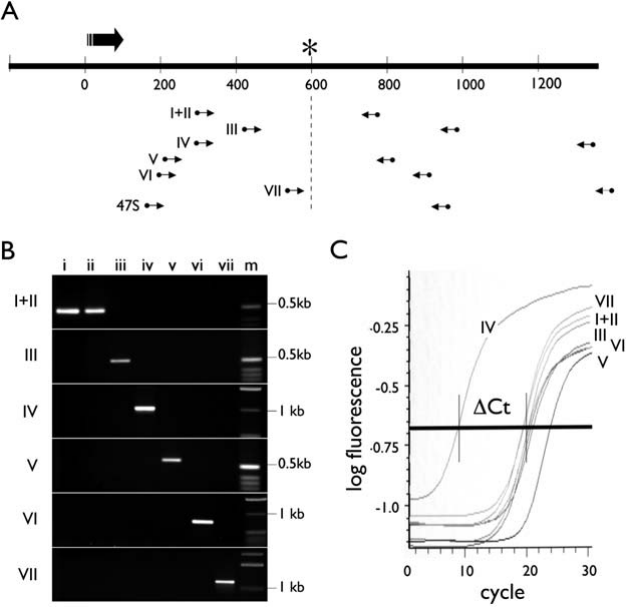
Variant-specific PCRs. A, Locations of the amplicons are shown in reference to the 5′-ETS. The large arrow depicts transcription start site and direction; asterisk, the first cleavage site in processing the pre-rRNA; small arrows, PCR primers; and the Roman numerals, PCR specificity. B, The specificity of each variant-specific PCR was tested with cloned v-rDNAs. The PCR was also adjusted, using cloned v-rDNA, to have similar amplification efficiency. Lowercase letters on top of the gel image indicate cloned templates, and uppercase letters to the left of image, the specificity of primers, m, molecular weight marker. C, An example of the specificity of qPCR for variant IV is shown (SYBG fluorescence). The delta Ct between specific and non-specific reactions is greater than 10.

Multi-sequence alignment showed variant-specific SNPs in the transcribed region of v-rDNA (**Fig. 3B**). Statistical analysis of the SNPs in the 26 v-rDNA clones confirmed the specificity of these SNPs and suggested that by employing two such SNPs, highly specific PCR assays could be developed for each v-rDNA type (**Table 2**). Based on these observations, we developed a set of variant-specific PCR assays. The variant-specific SNPs were examined for their specificity and location within the transcribed region. Six sets of SNPs were chosen to design the PCR primers (**Table 2**, **Fig. 5A** and Methods S1). In these PCR primers, the SNPs were the 3′ nucleotide matching to the sequence of its targeted v-rDNA. An exception was that for the 3′ primer of v-rDNA III, the specific nucleotide was in the middle of the primer (Methods S1). The primers were also selected to flank the first excision site of the pre-rRNA [34] to ensure amplification of only the full-length transcript (**Fig. 5A**). Using the cloned v-rDNA as templates, the PCR conditions were optimized so that each reaction amplified its intended target v-rDNA with high specificity (**Fig. 5B**). The sequences of v-rDNA I and II were very closely related and no primer could distinguish them and these two v-rDNA types were assayed together. Using the cloned v-rDNA as templates, the efficiencies of the variant-specific PCRs were adjusted to be similar. Some of these reactions (i.e., I+II, IV, V, VI) were adapted for a real-time PCR assay by adding SYBR green to the PCR reaction (**Fig. 5C**). Other reactions (i.e., III and VII) could not be adapted because their specificity deteriorated in the presence of SYBR, i.e., delta-Ct value for specific and non-specific templates was less than 10.

**Table 2:** Variant-specific single nucleotide polymorphism.

Type	clone	I-5*	I-3	III-5	III-3	IV-5	IV-3	V-5	V-3	VI-5	VI-3	VII-5	VII-3
I	I-A	**A**	**T**	A	C	A	T	G	T	G	C	C	T
	I-B	**A**	**T**	A	C	A	T	G	T	G	C	C	T
	I-C	**A**	**T**	A	C	A	T	G	T	G	C	C	T
	I-D	**A**	**T**	A	C	A	T	G	T	G	C	C	T
	I-F	**A**	**T**	A	C	A	T	G	T	G	C	C	T
	I-D	**A**	**T**	A	C	A	T	G	T	G	C	C	T
II	II-A	**A**	**T**	A	C	A	T	G	T	G	C	C	T
	II-B	**A**	**T**	A	C	A	T	G	T	G	C	C	T
III	III-A	A	C	**C**	**T**	A	T	G	T	G	C	C	T
	III-B	A	C	**C**	**T**	A	T	G	T	G	C	C	T
	III-C	A	C	**C**	**T**	A	T	G	T	G	C	C	T
	III-D	A	C	**C**	**T**	A	T	G	T	G	C	C	T
IV	IV-A	G	C	A	C	**G**	**G**	G	T	G	C	C	T
	IV-B	A	C	A	C	**A**	**G**	G	T	G	C	C	T
	IV-C	G	C	A	C	**G**	**G**	G	T	G	C	C	T
	IV-D	G	C	A	C	**G**	**G**	G	T	G	C	C	T
V	V-A	G	C	A	C	G	T	**T**	**C**	T	A	C	T
	V-B	G	C	A	C	G	T	**T**	**C**	T	A	C	T
VI	VI-A	G	C	A	C	A	T	G	T	**T**	**C**	C	T
	VI-B	A	C	A	C	T	T	G	T	**T**	**C**	C	T
	VI-C	A	C	A	C	A	T	T	T	**T**	**C**	C	T
	VI-D	G	C	A	C	G	T	T	T	**T**	**C**	C	T
VII	VII-A	A	C	A	C	G	T	T	T	G	C	**T**	**C**
	VII-B	T	C	A	C	A	T	T	T	G	C	**T**	**C**
	VII-C	G	C	A	C	A	T	G	T	G	C	**T**	**C**
	VII-D	A	C	A	C	T	T	G	T	G	C	**T**	**C**
	total	26	26	26	26	26	26	26	26	26	26	26	26
	A	17	0	22	0	17	0	0	0	0	2	0	0
	C	0	18	4	22	0	0	0	2	0	24	22	4
	G	8	0	0	0	7	4	20	0	20	0	0	0
	T	1	8	0	4	2	22	6	24	6	0	4	22
***p*** **-value**	**single**	1.6E-02	6.4E-07	6.7E-05	6.7E-05	4.5E-02	6.7E-05	4.6E-02	3.1E-03	1.0E-03	7.1E-01	6.7E-05	6.7E-05
	**double**	**6.4E-07**		**6.7E-05**		**5.9E-03**		**3.1E-03**		**6.7E-05**		**6.7E-05**	

* The SNPs are named according to their use in the variant-specific PCR assay (e.g., V-5 means that the SNP is used in the 5′ primer for assaying v-rDNA V). V-rDNAs I and II are assayed by the same pair of primers.

### Expression profile of v-rDNA in the mouse tissues

**Figure 6:**
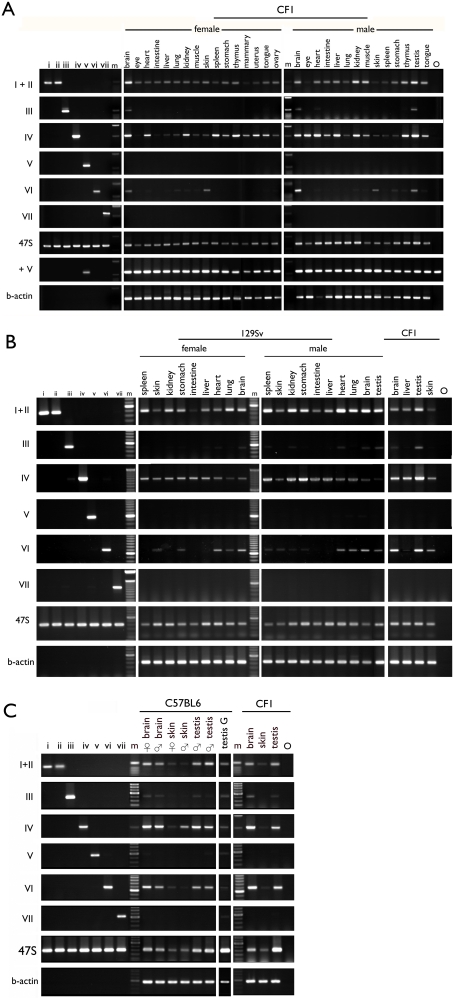
Expression of v-rDNA in mouse tissues. Variant-rDNA expression was assessed by RT-PCR in mouse strain CF1, (A), 129Sv, (B) and C57BL/6J, (C). A, RNAs from common and gender-specific tissues of one female and one male CF1 mice were assayed as indicated above the gel image. For a given tissue sample, all variant-specific PCRs were performed with the same cDNA preparation. The primer specificity is indicated on the left of the gel image. For each PCR, a set of controls was included to monitor the specificity (the left seven lanes) and the relative quantity and integrity of the RNA preparation (beta-actin). To assure no toxicity was present to prevent amplification of v-rDNA V, the cloned DNA was mixed with cDNA preparation before PCR (row +V). Similar results were obtained for VII (not shown). A PCR detecting all v-rDNA transcript (47S) is also included. m, molecular weight markers, O, blank. B and C, as in A except that each assay included a set of CF1 RNA samples as a reference (the last three or four lanes).

We used the variant-specific PCR assay to assess the expression level of each v-rDNA (**Fig. 6A**). Total RNA was prepared from common or gender-specific tissues of unrelated male and female CF1 mice. Based on semi-quantitative RT-PCR (random priming), the level of expression of the seven v-rDNAs could be classified into three groups. Variants I, II and IV were expressed universally (i.e., in all tissues examined), but the expression level of IV appeared to vary significantly in different tissues, suggesting expression modulation. In contrast, transcripts of V and VII were never detected in the 30 tissues examined. Interestingly, transcripts from III and VI were detected only in a limited number of tissues (**Fig. 6A**). Overall, most tissues contained three v-rDNAs (i.e., I, II and IV), but brain and testis contained five (i.e., I, II, III, IV and VI). Similar v-rDNA expression profiles were detected in individuals from mouse strains 129Sv and C57BL/6 (**Fig. 6B and C**), suggesting this profile was not individual- or strain-specific.

**Figure 7:**
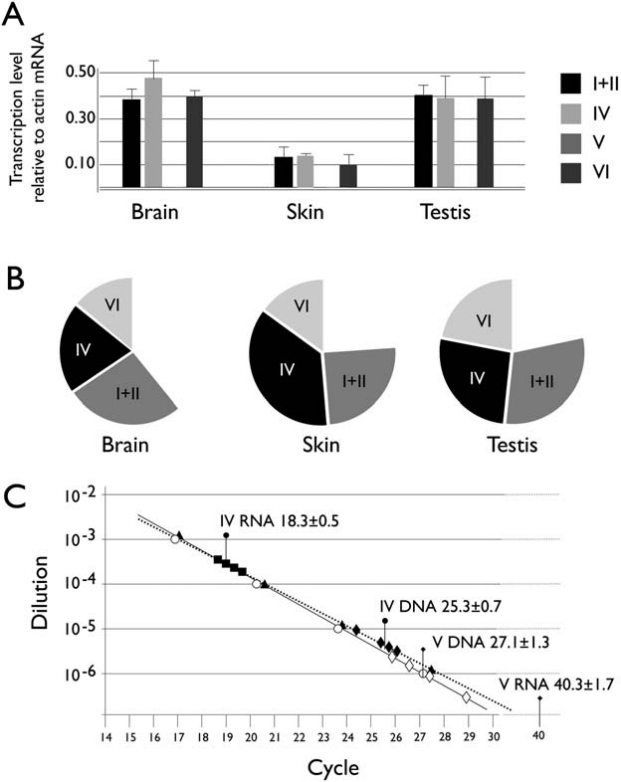
Quantitative analysis of v-rDNA expression. A, RNA samples from two hbyrids (C57BL/129Sv) were assayed with qPCR, the expression level was normalized with the transcript of beta-actin. (n = 2). Note that because V was not detected, only three bars are visible. B, In another experiment, the v-rDNA expression level was normalized with the 47S pre-rRNA (represented by the full circle) to show the contribution of each v-rDNA to the pre-rRNA pool. The values are average of two measurements. The blank sector is the portion of pre-rRNA not accounted for by the v-rDNA transcripts. C, A comparison of RNA and DNA quantity ratios. Ct values of RNA and DNA are superimposed on the standard curves of the two qPCRs for v-rDNA IV and V. Ct values of serial dilutions of cloned DNA (IV, black triangles; and V, open circles) are shown with the standard curve. RNA (transcript) Ct values are shown by squares (IV, black). The value of v-rDNA V is out of range, thus not plotted, but the average and standard deviation are indicated. Genomic DNA measurements are depicted as diamonds (IV, black and V, open). The average and standard deviation of 2 samples, each measured twice, are indicated by pins; circle-ended pins for variant IV and diamond-ended ones, V.

Using quantitative PCR (qPCR), we investigated the relative transcript level of each v-rDNA in brain, skin, and testis (**Fig. 7A**). The relative level of rDNA expression was higher in the brain and testis than that of skin. This study also suggested that in these three tissues, each v-rDNA contributed similarly to the pre-rRNA pool. This conclusion was confirmed when the level of v-rDNA transcripts was normalized against the level of the 47S pre-rRNA. Interestingly, the sum of the five v-rDNAs (i.e., I, II, IV, V and VI) could account for no more than 75% of the 47S pre-rRNA. Of the two v-rDNAs (i.e., III and VII) that were not measured because of technical difficulties, III was expressed at a low level and VII was not expressed at all (**Fig. 6**). Thus neither v-rDNA could make up the missing 25% of the pre-rRNA transcript. This observation suggested contributions from yet identified v-rDNAs.

We also investigated the relationship between expression level and gene copy number of variants IV and V in the testes of 129Sv/C57BL-6J hybrid mice. Variant IV was highly expressed whereas V was virtually silent. Furthermore, their PCR assays had similar amplification efficiency (**Fig. 7C**). Testes of two 129Sv/C57BL-6J mice were isolated; one of the testes from each mouse was used for RNA extraction and the other for genomic DNA isolation. Quantitative PCR showed that the average Ct value for v-rDNA IV transcript was 18.3±2.1 and that for v-rDNA V was greater than 40 (below background), which translated into at least 2^21.7^ or 3.7×10^6^-fold difference. The same assay showed that the Ct value of genomic v-rDNA IV was 25.3±2.1 and that of v-rDNA V 27.1±2.4, an approximately 4-fold difference (2^1.8^ = 3.48). The ratio of Ct values of RNA and DNA suggested that for each copy of v-rDNA IV there were 128 transcripts (i.e., 47S pre-rRNA), whereas no transcript was detected for v-rDNA V. This result demonstrated that the variations in expression level could not be explained by copy number alone, and thus differential transcription modulation or post-transcription processing should be considered.

## Discussion

Based on the data presented here, we propose that mouse rDNA array contains several variant types. The expression of each variant appears to be regulated independently from others and for some variants (i.e., III and VI), expression is tissue-specific. We describe several lines of evidence to support this proposal. Guided by the stable rDNA RFLPs, we cloned seven v-rDNAs; copy number analyses suggest that most variants contain between 8–15 copies; we identified variant-specific SNPs in the transcribed sequence and variant-specific PCR revealed variable expression of these v-rDNAs in different tissues; and v-rDNA expression profiles are conserved within and among mouse strains.

Our results are consistent with previous reports on the v-rDNA RFLPs. A previous study used two restriction enzymes (Eco RI and Hind III) to examine six inbred CBA/H-T6 individuals and found no variation in their rDNA RFLPs [8]. In addition, our study detected no variation among tissues of an individual (**Fig. 1B**). Taken together, these results indicate that non-homologous sequence exchanges (homogenization) among different mouse v-rDNAs are rare [17]. By molecular cloning of seven rDNA RFLPs, we were able to measure their genomic copy numbers. This measurement suggests that the v-rDNAs described here are a subset of the mouse rDNA array. Measurement of the intensity of hybridization signal indicated consistently a total copy number of between 50 to 80, which falls short of the estimated several hundreds copies of rDNA in mouse [35]. This discrepancy may in part be explained by the inherent difficulty in assessing copy numbers by hybridization, which depends on sequence homology. Any sequence variation between the probe and targets may result in reduced hybridization, and hence an underestimation of the copy number. Furthermore, our method also favors detecting rDNA configured as tandem repeats and likely underestimates rDNA in other configurations (e.g., single copy of varying size or inverted repeats), which are present in the mammalian rDNA array [14], [15].

The DNA methylation status of the v-rDNA also suggests that we are dealing with a subset of rDNA. DNA methylation measurements by HpaII sensitivity as well as by bisulfite sequencing showed that the promoters of the seven v-rDNAs are hypomethylated, whereas the same method detected hypermethylated rDNA promoters in the genome. Furthermore, bisulfite sequencing suggested that ∼17% of the mouse genomic rDNA promoters are hypomethylated (**Fig. 4D, E**). Taken together, if these hypomethylated rDNAs obtained from random samplings are the same that we obtained by RFLP-based cloning (i.e., the 50–80 copies of v-rDNAs), then the total copy number of rDNA in the mouse genome would be between 294 to 471, which is remarkably close to the estimated 300 to 400 copies of rDNA in mouse. This and other analyses discussed below strongly suggest that we are dealing with a subset of rDNA.

A related question is why we isolated only hypomethylated rDNA. We speculate that our method can only detect v-rDNA arrays that are arranged as tandem repeats (i.e., yielding a single DNA fragment upon restriction digestion). On the other hand, if the methylated rDNAs are in a different configuration or their restriction fragment length varies from unit to unit, then restriction digestion will produce a smear of bands on the gel, which would not be identified and cloned by our method. A recent study of human rDNA array did show rDNA units of variable length, as well as existing in configurations other than tandem repeats (e.g., single copy outside rDNA clusters, or inverted repeat) [14], [15]. Our speculation is only one of several possible explanations.

The copy number analysis also suggests that each type of v-rDNA contains between 8–15 copies, which are arranged in tandem (i.e., yielding restriction fragments of the same size). An ambiguity is that we could not measure by Southern blot the copy number of v-rDNA IV and V separately. However, qPCR measurements suggest that their copy number ratio is 3.48 (**Fig. 7C**). Because their combined copy number measured by Southern blot is on average ∼16, this translates into that 12 IV copies and 4 V copies. This estimate makes V the least abundant v-rDNA, which is consistent with our isolating only two clones from a total of 33 v-rDNA clones. Thus it appears that v-rDNAs are blocks of tandem repeats of no more than 20 units. Whether this block size is due to a limitation in stability of tandem repeats or the regulatory requirement of rDNA remains to be investigated. It is reasonable to assume that if each v-rDNA type is a cluster of tandem repeats of no more than 20 copies, then the cluster is very likely located at one chromosome locus.

The expression studies suggest that the v-rDNA types are regulated differentially. The constitutively expressed v-rDNAs (I, II and IV) represented approximately 56% of the copies of the v-rDNA detected. The selectively expressed III and VI constitute about 29% copies, in which VI is expressed more widely than III, suggesting that they are individually regulated. The silent V and VII have ∼15% of the copies. Our data show that the three v-rDNA regulatory groups are present in six mice of three strains, which strongly suggest that their regulation is not randomly determined. It should be noted that our analysis employs tissues containing many cell types, each of which may have a unique profile of v-rDNA expression. When assayed as a mixture, the unique v-rDNA profile of each cell type may be obscured (i.e., being diluted or canceled out). Furthermore, our observation is limited developmentally to a narrow window in adulthood. Therefore, our results are likely an underestimate of the complexity of v-rDNA regulation. This point is of particular relevance in interpreting the apparent lack of expression of v-rDNA V and VII. It remains possible that these v-rDNAs are expressed in a small number of cells or at a particular developmental stage, and thus not detected by our screen. Consistent with this proposal is that v-rDNA VII is expressed in corneal epithelial keratinocytes [25]. Nevertheless, the over all regulatory patterns are clear and indicate cell/tissue-specific regulation of rRNA synthesis. Most important, these results suggest that mammals possess regulatory mechanisms that determine precisely which v-rDNA to express in different tissues.

Although our data do not discern how the regulation is achieved (i.e., transcription or/and processing, or by yet unidentified mechanisms) for the actively transcribed rDNA (i.e., hypomethylated), a number of transcriptional and processing regulatory mechanisms have been reported (for recent reviews, [35]–[40]. It is possible that these mechanisms can exert their effect on a selective subset of rDNA, but that has not been demonstrated. These mechanisms are likely responsible for regulation of constitutively expressed v-rDNA. The question then becomes what mechanism is responsible for the regulation of cell/tissue-specific expression. Although our data do not provide a definitive answer, they do suggest reasons for eliminating several possibilities. (i) We noted that SNPs in the rDNA promoters appear to correlate with their activity, e.g., Class I (G at −179) correlates with constitutive expression and Class 2 (A at −219) correlates with selective expression. However, these SNP markers are also present in hypermethylated rDNA promoters, which are transcriptionally silent. Therefore, the SNPs cannot be the sole determinants of promoter activity. (ii) Copy-number is unable to account for the variation of v-rDNA transcript level in different tissues. The variants III and IV are present in similar copy numbers, but III is selectively, whereas IV constitutively, expressed. (iii) Furthermore, the variable expression pattern of v-rDNA among different tissues is unlikely the result of promoter exchange via recombination between non-homologues rDNA loci during organogenesis. This conclusion is based on our observation that rDNA RFLPs are invariable among tissues of the same individual. (iv) Mouse rDNA is partitioned into transcriptionally active and silent blocks by epigenetic modifications (i.e. DNA methylation and chromatin modification) [41]–[47]. We detect in the v-rDNA VII promoter (silent) cytosine methylation of CpG at −133, which was shown to block transcription [45]. This mechanism may explain the silence of VII in most tissues. However, DNA methylation cannot explain the tissue-specific expression of III and VI, because their promoters are hypomethylated in both liver and testis, but their expression is primarily detected only in the latter. (v) The variable number of enhancers, which stimulates Pol I transcription [48]–[50], may account for the silence of VII (3–4 enhancer units) and the selective activity of VI (20 units). Nevertheless, this parameter alone cannot explain the differential activity of IV (constitutively active) and V (silent), which have similar numbers of enhancer units (10 and 11). This analysis suggests yet unidentified mechanisms in cell-type- and v-rDNA-specific regulation.

Our findings raise the question regarding the biological significance of cell-type-specific regulation of v-rDNA, and the developmental-stage-specific regulation of variant rRNA noted previously in malaria parasites Plasmodium [51], [52]. In mammals, our finding is consistent with the “ribosome filter hypothesis” [53], [54], which is based on observations that many mRNAs contain sequence similar or complementary to the 18S rRNA. The ribosome filter hypothesis proposes that differential binding of particular mRNAs to eukaryotic 40S ribosomal subunits before translation may selectively affect rates of translation. In this view, ribosomal subunits themselves are considered regulatory elements or filters that mediate interactions between particular mRNAs and components of the translation machinery. These interactions would depend, in part, on the complementarity between sequences in mRNA and rRNA, as well as on structural differences among ribosomes in different cell types. Thus, a major predication of the ribosome filter hypothesis is that rRNA population is heterogeneous and different rDNAs are expressed with cell-type-specificity.

Our results support this prediction. The biological meaning of cell-type-specific regulation of v-rDNA is also hinted from the function of BNC1, which regulates a subset of rDNA [24], [25]. Using a BNC1 knock-down model, we showed that *Bnc1* is a mammalian maternal-effect gene, i.e., embryos derived from BNC1-deficient oocytes die at the 2-cell stage [23]. The maternal effect of BNC1 is of particular interest because oocytes synthesize and accumulate a large amount of rRNA during their growth, presumably for use in early embryos, which depend entirely on maternal ribosomes for their translational need [55]–[59]. It is therefore paradoxical that in mouse a substantial amount of maternal rRNA and ribosome are degraded during oocyte maturation prior to fertilization [60], [61]. This paradox is rooted in the dogma of ribosome biology that for each species one type of ribosome suffices. It is tempting to speculate that cell-type-specific regulation of v-rDNA represents a differential requirement of subtypes of ribosomes during development, e.g., upon maturation, mouse oocytes degrade the ribosomes not required or detrimental for embryonic development. This interpretation can also explain BNC1's maternal effect; BNC1 promotes production of a subtype of ribosomes required for early embryonic development.

In summary, using rDNA RFLP, we identified seven rDNA variants. Each variant appears to contain approximately 10 to 15 transcription units arranged as tandem repeats. The promoters of the seven variant types are hypomethylated, which suggests that they are transcriptionally active. We show by variant-specific PCRs that these v-rDNAs are not regulated in concert, but independently, and in some cases, tissue-specifically. Our results provide the first molecular evidence of tissue-specific usage of a subset of rDNA. In light of recent findings that human rDNA transcription units are variable in length and arranged in a variety of configurations [14], [15], our observations also suggest heretofore-unappreciated complexity in mouse rDNA structure and regulation.

## Materials and Methods

*Mouse Strains* CF1, 129Sv and C57BL/6J mice were purchased from Charles River Laboratory and sacrificed on arrival. C57BL/6J/129Sv hybrid mice were F4 progeny of a backcross of 129Sv:C57BL×C57BL [25]. The exact genetic contribution from each strain was not well defined. The mouse experiments were performed according to the protocols approved by University of Pennsylvania Animal Care and Use Committee.

### Southern analysis and the cloning of v-rDNA

Genomic DNA isolation, restriction digestion, Southern analysis, construction of libraries of size-fractionated genomic DNA and colony hybridization were performed as previously described [63]. The Southern probes were prepared by PCR using primers derived from published sequences (GenBank BK000964 and X82564) [19], [62]. Each blot was analyzed by comparing the size of the hybridizing fragments with that predicted by the known sequence (**Fig. 1A, B**). To reduce the chance of partial digestion, genomic DNAs were over-digested by more than 50-fold (i.e., the amount of enzyme and the duration of digestion were sufficient to digest 50-fold more substrate DNA). All hybridizations were done at high stringency (i.e., hybridization at 65°C in 1M NaCl and final wash at 0.2×SSC at 65°C).

### RNA analysis (RT-PCR and qPCR)

RNA isolation and RT-PCR analysis were performed as previously described [64]. Variant-specific primers and PCR conditions (primers and cycling parameters) are provided in the supplementary materials (Methods S1. Quantitative PCRs were done first on a DNA Engine OPTICON2 (MJ Research, MA) with a kit (F-400RS/L, Finnzyme Oy, Espoo, Finland), and repeated on an Applied Biosystems 7500 Real Time PCR, following manufacturers' instructions. Beta-actin mRNA and 47S pre-rRNA were used as internal reference.

*DNA sequencing* was performed at PENN DNA Sequencing Facility. Bisulfite sequencing was done with an EZ DNA methylation Kit (ZYMO Research Co., Orange, CA) following manufacturer's instructions. Sequence alignment was first performed by the computer program Clustal-W implemented in Vector NTI (Invitrogen) or as a web-based program (http://bioinfo.genopole-toulouse.prd.fr/multalin/) [65]. The machine alignments were then refined manually.

## Supporting Information

Table S1 Genbank accession numbers of v-rDNA sequences
(0.02 MB DOC)

Methods S1 (3.67 MB PDF)
