# Supplementary material for: Mouse Ribosomal RNA Genes Contain Multiple Differentially Regulated Variants
Source: PLoS One. 2008 Mar 26;3(3):e1843. doi: 10.1371/journal.pone.0001843 (PMC2266999; doi:10.1371/journal.pone.0001843)
Supplement: Table S1 — Genbank accession numbers of v-rDNA sequences (0.02 MB DOC) [file pone.0001843.s001.doc]

Table S1. Genbank Accession numbers of the v-rDNA sequences (nucleotides 1-2000).

vrDNA-1A EU433294

vrDNA-1B EU433295

vrDNA-1C EU433296

vrDNA-1D EU433297

vrDNA-1E EU433298

vrDNA-1F EU433299

vrDNA-2A EU433300

vrDNA-2B EU433301

vrDNA-3A EU433302

vrDNA-3B EU433303

vrDNA-3C EU433304

vrDNA-3D EU433305

vrDNA-4A EU433306

vrDNA-4B EU433307

vrDNA-4C EU433308

vrDNA-4D EU433309

vrDNA-5A EU433310

vrDNA-5B EU433311

vrDNA-6A EU433312

vrDNA-6B EU433313

vrDNA-6C EU433314

vrDNA-6D EU433315

vrDNA-7A EU433316

vrDNA-7B EU433317

vrDNA-7C EU433318

vrDNA-7D EU433319

Nomenclature: the number in the name indicates the subtyppe (i.e., I to VII) and the letter indicates a randomly picked clone, e.g., vrDNA-1A means clone A of vrDNA subtype I.
