# Supplementary material for: Mouse Ribosomal RNA Genes Contain Multiple Differentially Regulated Variants
Source: PLoS One. 2008 Mar 26;3(3):e1843. doi: 10.1371/journal.pone.0001843 (PMC2266999; doi:10.1371/journal.pone.0001843)
Supplement: Methods S1 — (3.67 MB PDF) [file pone.0001843.s002.pdf]

Supplement Methods
Variant-rDNA-specific PCR

No SNP was identified to distinguish v-rDNA I and II (i.e. their sequences were identical within the sequenced region of 5'-ETS). They were therefore always assayed together. For a given tissue sample, all PCRs were performed with the same cDNA preparation.

Regular PCR

v-rDNA	primers	(1X) buffer	thermal cycle
I	5'-cca gct gtg gtt gag ggc c**a**<br>5'-cgc tgg cag aac gag aag a**a**	10 mM Tris-HCl, pH 8.8; 1.5 mM $MgCl_2$; 75 mM KCl	94°C 5 m;{94°C, 30 s; 69°C, 30 s; 72°C, 40 s} x 35; 72°C, 7 m
II	Same as I	Same as I	Same as I
III	5'-ccg agt **ac**t tct cct gtc tg<br>5'-gcc acc ggc cac atc cac c**a**	Same as I	94°C 5 m;{94°C, 30 s; 66°C, 30 s; 72°C, 1.5 m} x 35; 72°C, 7 m
IV	5'-cca gct gtg gtt gag ggc c**g**<br>5'-aag ctc ccc acg gga aag c**c**	Same as I	94°C 5 m;{94°C, 30 s; 65°C, 30 s; 72°C, 1.2 m} x 35; 72°C, 7 m
V	5'-gtg act ttg cgt gtc aga **ct**<br>5'-aca cac cac cgg cag acg **gg**	Same as I	94°C 5 m;{94°C, 30 s; 68°C, 30 s; 72°C, 1.5 m} x 35; 72°C, 7 m
VI	5'-gtg act ttg cgt gtc aga c**t**<br>5'-aca cgt gag ggc aca acc g**g**	Same as I	94°C 5 m;{94°C, 30 s; 67°C, 30 s; 72°C, 40 s} x 40; 72°C, 7 m
VII	5'-ctc ttg ttc tgt gtc tgt a**t**<br>5'-gat ccc tcc ccg aac tcg g**g**	10 mM Tris-HCl, pH 8.3; 3.5 mM $MgCl_2$; 25 mM KCl	94°C 5 m;{94°C, 30 s; 62°C, 30 s; 72°C, 1.5 s} x 35; 72°C, 7 m
47S	5'-tgt gac aac tgg gcg ctg tg<br>5'-cac tga gaa aag tgc gcg cg	Same as I	94°C 5 m;{94°C, 30 s; 66°C, 30 s; 72°C, 40 s} x 35; 72°C, 7 m

Real Time PCR

v-rDNA	primers	Reaction mix	thermal cycle
I	5'-cca gct gtg gtt gag ggc c**a**<br>5'-cgc tgg cag aac gag aag a**a**	10 microliters master mix, 5 microliters of primers and 5 microliters of template	95°C 10 m;{94°C, 10 s; 68°C, 15 s; 72°C, 40 s, read} x 35, 72°C, 10 m
II	Same as I	Same as I	Same as I
III	5'-ccg agt **ac**t tct cct gtc tg<br>5'-gcc acc ggc cac atc cac c**a**	Same as I	95°C 10 m;{94°C, 10 s; 66°C, 15 s; 72°C, 80 s, read} x 45, 72°C, 10 m
IV	5'-cca gct gtg gtt gag ggc c**g**<br>5'-aag ctc ccc acg gga aag c**c**	Same as I	95°C 10 m;{94°C, 10 s; 65°C, 15 s; 72°C, 40 s, read} x 45, 72°C, 10 m
V	5'-gtg act ttg cgt gtc aga **ct**<br>5'-aca cac cac cgg cag acg **gg**	Same as I	95°C 10 m;{94°C, 10 s; 68°C, 15 s; 72°C, 40 s, read} x 35, 72°C, 10 m
VI	5'-gtg act ttg cgt gtc aga c**t**<br>5'-aca cgt gag ggc aca acc g**g**	Same as I	95°C 10 m;{94°C, 10 s; 66°C, 15 s; 72°C, 40 s, read} x 45, 72°C, 10 m
VII	5'-ctc ttg ttc tgt gtc tgt a**t**<br>5'-gat ccc tcc ccg aac tcg g**g**	Same as I	95°C 10 m;{94°C, 10 s; 62°C, 15 s; 72°C, 40 s, read} x 45, 72°C, 10 m
47S	5'-tgt gac aac tgg gcg ctg tg<br>5'-cac tga gaa aag tgc gcg cg	Same as I	95°C 10 m;{94°C, 10 s; 66°C, 15 s; 72°C, 40 s, read} x 35, 72°C, 10 m

The bold letters in primer sequence indicates the SNPs that are specific to the v-rDNA subtype.
